# 1,3-Diprop-2-ynyl-1*H*-imidazol-3-ium bromide

**DOI:** 10.1107/S1600536808010726

**Published:** 2008-04-23

**Authors:** Hui Li, Lin-Yu Jin, Ruo-Jie Tao

**Affiliations:** a Institute of Molecular and Crystal Engineering, College of Chemistry and Chemical Engineering, Henan University, Kaifeng 475001, Henan, People’s Republic of China; b College of Chemistry and Chemical Engineering, Henan University, Kaifeng 475001, Henan, People’s Republic of China

## Abstract

In the title salt, C_9_H_9_N_2_^+^·Br^−^, the ethynyl groups are nearly anti­parallel to each other [the angle between the two ethynyl groups is179.7 (2)°]. No classical hydrogen bonds or π–π inter­actions are observed. The mol­ecules are linked by C—H⋯Br hydrogen bonds. The bromide anions are involved in inter­actions with three H atoms.

## Related literature

For related literature, see: Fei *et al.* (2004 ▶); Rajesh *et al.* (2008 ▶).
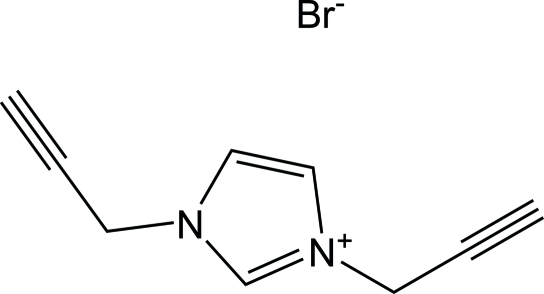


## Experimental

### 

#### Crystal data

C_9_H_9_N_2_^+^·Br^−^
*M* *_r_* = 225.09
Monoclinic, 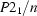

*a* = 8.3439 (8) Å
*b* = 12.1069 (11) Å
*c* = 10.0413 (9) Å
β = 112.263 (2)°
*V* = 938.74 (15) Å^3^
*Z* = 4
Mo *K*α radiation
μ = 4.32 mm^−1^
*T* = 273 (2) K
0.18 × 0.16 × 0.15 mm

#### Data collection

Bruker  SMART APEXII CCD area-detector diffractometer
Absorption correction: none
4482 measured reflections
1650 independent reflections
1580 reflections with *I* > 2σ(*I*)
*R*_int_ = 0.020

#### Refinement

*R*[*F* ^2^ > 2σ(*F* ^2^)] = 0.019
*wR*(*F* ^2^) = 0.051
*S* = 1.08
1650 reflections
109 parameters
H-atom parameters constrained
Δρ_max_ = 0.48 e Å^−3^
Δρ_min_ = −0.37 e Å^−3^

### 

Data collection: *APEX2* (Bruker, 2005 ▶); cell refinement: *APEX2*; data reduction: *SAINT* (Bruker, 2005 ▶); program(s) used to solve structure: *SHELXS97* (Sheldrick, 2008 ▶); program(s) used to refine structure: *SHELXL97* (Sheldrick, 2008 ▶); molecular graphics: *SHELXTL* (Sheldrick, 2008 ▶); software used to prepare material for publication: *SHELXTL*.

## Supplementary Material

Crystal structure: contains datablocks I, global. DOI: 10.1107/S1600536808010726/fb2094sup1.cif

Structure factors: contains datablocks I. DOI: 10.1107/S1600536808010726/fb2094Isup2.hkl

Additional supplementary materials:  crystallographic information; 3D view; checkCIF report

## Figures and Tables

**Table 1:** Hydrogen-bond geometry (Å, °)

*D*—H⋯*A*	*D*—H	H⋯*A*	*D*⋯*A*	*D*—H⋯*A*
C9—H9*B*⋯Br1^i^	0.97	2.75	3.6748 (19)	159
C8—H8⋯Br1^ii^	0.93	2.81	3.7105 (19)	164
C6—H6*B*⋯Br1^iii^	0.97	2.81	3.7196 (18)	157

Symmetry codes: (i) 
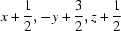
; (ii) 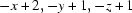; (iii) 
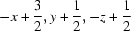
.

**Fig. 1.**
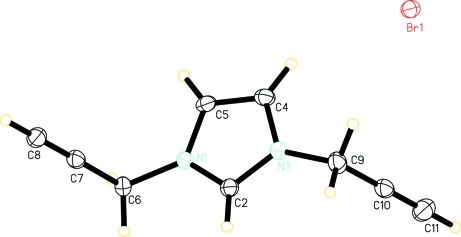
The title molecule with the atom-labelling scheme. The displacement ellipsoids are drawn at the 50% probability level.

**Fig. 2.**
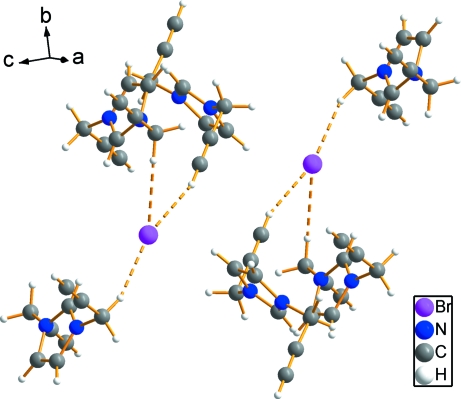
The crystal structure of the title compound. The C—H···Br hydrogen bonds are indicated by the dashed lines.

**Fig. 3.**
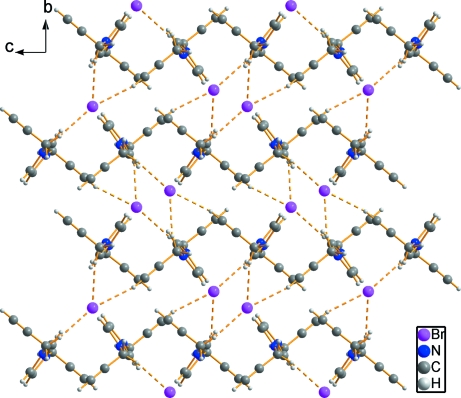
The molecular packing of the title compound viewed along the a axis. The hydrogen bonds are indicated by dashed lines.

## supplementary crystallographic information

### Comment

The constituing molecule of the title compound is shown in Fig. 1. Both ethynyls in the title molecule are nearly antiparallel to each other [the angle equals to 179.7 (2)°]. Except for each ethinyl, all the remaining non-H atoms are almost coplanar, with a mean deviation from the least-square plane to be 0.006 (1)Å. The angles between each ethinyl and this plane are about equal [26.8 (1) and 26.3 (1)°]. The bond lengths and angles are normal.

The molecules are linked by C—H···Br hydrogen bonds. Each Br atoms is involved in the C—H···Br interaction with three hydrogens. One of these hydrogens is the ethinyl hydrogen while the remaining two stem from the methylene groups (Fig. 2). There are intermolecular C—H···Br hydrogen bonds in the structure (Fig. 3). No conventional hydrogen bond or π-π electron interactions have been observed.

### Experimental

A mixture of imidazole (0.6808 g, 0.01 mol) and propargyl bromide (2.379 g, 0.02 mol) in toluene was refluxed and stirred at room temperature for one day. The resulting solid was filtered, washed with diethyl ether and dried under vacuum for two days. X-ray-quality block-like crystals were grown by slow diffusion of N,N-dimethylformamide into a methyl alcohol solution of the title compound. Average size of the crystals was about 0.15 mm in each direction.

### Refinement

All the H atoms could be detected in the difference electron density maps. Nevertheless, they were situated into the idealized positions and refined using a riding model. C—H = 0.97 Å for the methylene groups and C—H = 0.93 Å for the remaining H atoms. *U*_iso_(H) = 1.2*U*_eq_(C) for all the H atoms.

### Crystal data

**Table:**

C_9_H_9_N_2_^+^·Br^–^	*F*_000_ = 448
*M**_r_* = 225.09	*D*_x_ = 1.593 Mg m^−^^3^
Monoclinic, *P*2_1_/*n*	Mo *K*α radiation λ = 0.71073 Å
Hall symbol: -P 2yn	Cell parameters from 4175 reflections
*a* = 8.3439 (8) Å	θ = 2.7–28.3º
*b* = 12.1069 (11) Å	µ = 4.32 mm^−^^1^
*c* = 10.0413 (9) Å	*T* = 273 (2) K
β = 112.263 (2)º	Block, colourless
*V* = 938.74 (15) Å^3^	0.18 × 0.16 × 0.15 mm
*Z* = 4	

### Data collection

**Table:**

Bruker SMART CCD area-detector diffractometer	1580 reflections with *I* > 2σ(*I*)
Radiation source: fine-focus sealed tube	*R*_int_ = 0.020
Monochromator: graphite	θ_max_ = 25.0º
*T* = 273(2) K	θ_min_ = 2.8º
φ and ω scans	*h* = −9→7
Absorption correction: none	*k* = −14→14
4482 measured reflections	*l* = −11→11
1650 independent reflections	

### Refinement

**Table:**

Refinement on *F*^2^	Secondary atom site location: difference Fourier map
Least-squares matrix: full	Hydrogen site location: difference Fourier map
*R*[*F*^2^ > 2σ(*F*^2^)] = 0.019	H-atom parameters constrained
*wR*(*F*^2^) = 0.051	*w* = 1/[σ^2^(*F*_o_^2^) + (0.0259*P*)^2^ + 0.4349*P*] where *P* = (*F*_o_^2^ + 2*F*_c_^2^)/3
*S* = 1.08	(Δ/σ)_max_ = 0.002
1650 reflections	Δρ_max_ = 0.49 e Å^−^^3^
109 parameters	Δρ_min_ = −0.37 e Å^−^^3^
36 constraints	Extinction correction: none
Primary atom site location: structure-invariant direct methods	

### Special details

**Table:**

Geometry. All e.s.d.'s (except the e.s.d. in the dihedral angle between two l.s. planes) are estimated using the full covariance matrix. The cell e.s.d.'s are taken into account individually in the estimation of e.s.d.'s in distances, angles and torsion angles; correlations between e.s.d.'s in cell parameters are only used when they are defined by crystal symmetry. An approximate (isotropic) treatment of cell e.s.d.'s is used for estimating e.s.d.'s involving l.s. planes.
Refinement. Refinement of *F*^2^ against ALL reflections. The weighted *R*-factor *wR* and goodness of fit *S* are based on *F*^2^, conventional *R*-factors *R* are based on *F*, with *F* set to zero for negative *F*^2^. The threshold expression of *F*^2^ > σ(*F*^2^) is used only for calculating *R*-factors(gt) *etc*. and is not relevant to the choice of reflections for refinement. *R*-factors based on *F*^2^ are statistically about twice as large as those based on *F*, and *R*- factors based on ALL data will be even larger.

### Fractional atomic coordinates and isotropic or equivalent isotropic displacement parameters (Å^2^)

**Table:**

	*x*	*y*	*z*	*U*_iso_*/*U*_eq_	
Br1	0.32729 (2)	0.540752 (13)	0.108115 (17)	0.01767 (9)	
N1	1.03714 (18)	0.71725 (12)	0.30495 (15)	0.0155 (3)	
C2	0.9295 (2)	0.77650 (14)	0.34460 (18)	0.0161 (3)	
H2	0.9582	0.8386	0.4034	0.019*	
N3	0.77349 (18)	0.73157 (12)	0.28557 (15)	0.0161 (3)	
C4	0.7818 (2)	0.64081 (14)	0.20458 (18)	0.0184 (4)	
H4	0.6906	0.5948	0.1518	0.022*	
C5	0.9476 (2)	0.63230 (14)	0.21711 (19)	0.0173 (4)	
H5	0.9932	0.5791	0.1745	0.021*	
C6	1.2230 (2)	0.73953 (14)	0.34599 (19)	0.0176 (4)	
H6A	1.2538	0.7292	0.2629	0.021*	
H6B	1.2468	0.8158	0.3768	0.021*	
C7	1.3294 (2)	0.66665 (14)	0.46228 (19)	0.0184 (4)	
C8	1.4191 (2)	0.60767 (15)	0.55419 (19)	0.0210 (4)	
H8	1.4897	0.5613	0.6265	0.025*	
C9	0.6171 (2)	0.77060 (16)	0.3052 (2)	0.0208 (4)	
H9A	0.5493	0.7074	0.3123	0.025*	
H9B	0.6507	0.8116	0.3946	0.025*	
C10	0.5102 (2)	0.84122 (15)	0.1865 (2)	0.0215 (4)	
C11	0.4197 (3)	0.89966 (16)	0.0955 (2)	0.0300 (4)	
H11	0.3482	0.9459	0.0235	0.036*	

### Atomic displacement parameters (Å^2^)

**Table:**

	*U*^11^	*U*^22^	*U*^33^	*U*^12^	*U*^13^	*U*^23^
Br1	0.01860 (13)	0.01479 (12)	0.01818 (13)	0.00031 (6)	0.00534 (9)	−0.00054 (6)
N1	0.0148 (7)	0.0148 (7)	0.0148 (7)	0.0009 (6)	0.0032 (6)	0.0016 (6)
C2	0.0166 (8)	0.0155 (8)	0.0138 (8)	0.0005 (7)	0.0030 (7)	0.0007 (7)
N3	0.0145 (7)	0.0164 (7)	0.0162 (7)	0.0005 (6)	0.0044 (6)	0.0005 (6)
C4	0.0208 (9)	0.0144 (8)	0.0168 (9)	−0.0019 (7)	0.0034 (7)	−0.0011 (7)
C5	0.0211 (9)	0.0121 (8)	0.0178 (9)	0.0013 (7)	0.0062 (7)	−0.0002 (7)
C6	0.0133 (8)	0.0182 (8)	0.0202 (9)	0.0006 (7)	0.0051 (7)	0.0006 (7)
C7	0.0160 (8)	0.0179 (8)	0.0206 (9)	−0.0014 (7)	0.0063 (7)	−0.0050 (7)
C8	0.0201 (9)	0.0193 (9)	0.0196 (9)	0.0025 (7)	0.0030 (7)	−0.0028 (8)
C9	0.0162 (8)	0.0241 (9)	0.0227 (9)	−0.0005 (7)	0.0080 (7)	−0.0025 (7)
C10	0.0173 (9)	0.0196 (9)	0.0272 (10)	−0.0023 (7)	0.0078 (8)	−0.0081 (8)
C11	0.0279 (10)	0.0232 (10)	0.0318 (11)	0.0052 (9)	0.0033 (9)	−0.0038 (9)

### Geometric parameters (Å, °)

**Table:**

N1—C2	1.323 (2)	C6—C7	1.464 (2)
N1—C5	1.376 (2)	C6—H6A	0.9700
N1—C6	1.471 (2)	C6—H6B	0.9700
C2—N3	1.326 (2)	C7—C8	1.183 (3)
C2—H2	0.9300	C8—H8	0.9300
N3—C4	1.384 (2)	C9—C10	1.463 (3)
N3—C9	1.470 (2)	C9—H9A	0.9700
C4—C5	1.345 (3)	C9—H9B	0.9700
C4—H4	0.9300	C10—C11	1.176 (3)
C5—H5	0.9300	C11—H11	0.9300
			
C2—N1—C5	109.43 (14)	C7—C6—H6A	109.3
C2—N1—C6	125.46 (14)	N1—C6—H6A	109.3
C5—N1—C6	125.09 (14)	C7—C6—H6B	109.3
N1—C2—N3	107.86 (15)	N1—C6—H6B	109.3
N1—C2—H2	126.1	H6A—C6—H6B	108.0
N3—C2—H2	126.1	C8—C7—C6	177.89 (19)
C2—N3—C4	109.20 (15)	C7—C8—H8	180.0
C2—N3—C9	125.44 (15)	C10—C9—N3	112.18 (15)
C4—N3—C9	125.35 (15)	C10—C9—H9A	109.2
C5—C4—N3	106.54 (15)	N3—C9—H9A	109.2
C5—C4—H4	126.7	C10—C9—H9B	109.2
N3—C4—H4	126.7	N3—C9—H9B	109.2
C4—C5—N1	106.97 (15)	H9A—C9—H9B	107.9
C4—C5—H5	126.5	C11—C10—C9	176.6 (2)
N1—C5—H5	126.5	C10—C11—H11	180.0
C7—C6—N1	111.66 (14)		
			
C5—N1—C2—N3	0.45 (18)	C2—N1—C5—C4	−0.31 (19)
C6—N1—C2—N3	179.45 (15)	C6—N1—C5—C4	−179.32 (15)
N1—C2—N3—C4	−0.41 (19)	C2—N1—C6—C7	101.28 (18)
N1—C2—N3—C9	178.53 (15)	C5—N1—C6—C7	−79.9 (2)
C2—N3—C4—C5	0.22 (19)	C2—N3—C9—C10	97.5 (2)
C9—N3—C4—C5	−178.72 (15)	C4—N3—C9—C10	−83.8 (2)
N3—C4—C5—N1	0.06 (19)		

### Hydrogen-bond geometry (Å, °)

**Table:**

*D*—H···*A*	*D*—H	H···*A*	*D*···*A*	*D*—H···*A*
C9—H9B···Br1^i^	0.97	2.75	3.6748 (19)	159
C8—H8···Br1^ii^	0.93	2.81	3.7105 (19)	164
C6—H6B···Br1^iii^	0.97	2.81	3.7196 (18)	157

Symmetry codes: (i) *x*+1/2, −*y*+3/2, *z*+1/2; (ii) −*x*+2, −*y*+1, −*z*+1; (iii) −*x*+3/2, *y*+1/2, −*z*+1/2.
